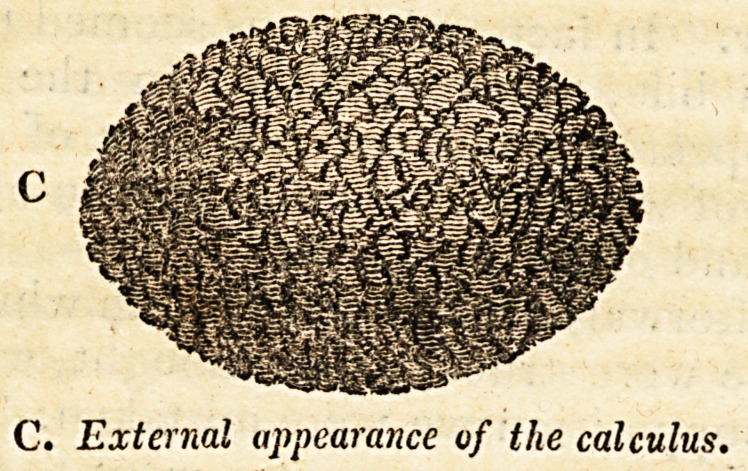# Case of Gastro-Enteritis, Terminating Fatally. With an Account of the Appearance of Some of the Abdominal Viscera after Death

**Published:** 1821-10

**Authors:** 


*Case of Gastro-enteritis, terminating fatally. With an Account of the Appearance of some of the Abdominal Viscera after Death.* By the Editor.

VARIOUS circumstances have lately combined to direct the attention of the medical profession to the irritative diseases of the mucous membranes, particularly of that of the stomach and intestines. The ravages committed by the intestinal fever of India have thrown on this subject an interest, which the observations of Broussais on Membranous Phlegmasiæ,—the Commentaries of Abercrombie, of Edinburgh, on Affections of the Intestines,—and the respectable works of some of the modern English writers on diseases of the stomach, have greatly contributed to heighten, and, we trust, to render permanent.

While it is acknowledged, on the one hand, that the application of theory to explain the many curious facts connected with the subject of inflammation of the mucous membranes, may lead to beneficial results; it cannot be denied, on the other hand, that too great a propensity to theorize, while the necessary data for a right induction are yet insufficient, must tend greatly to throw as much disrepute on this particular and important investigation, as a similar disposition of the mind, on the part of some medical men, has of late years been the means of bringing down on other equally interesting subjects of medical inquiry.

In this country, with the few exceptions just now alluded to, we collect facts, and are often guided by them. Sydenham taught us this lesson; which, disregarded, at first, by his immediate successors, is now pretty generally acted upon by the present generation of physicians.

In our practice we are greatly influenced by precedents; and, although it may be questioned how far such a method may, or may not, promote the advancement of medical knowledge; it is a fact which can be almost mathematically demonstrated, that, in a long series of years, the *benefit* of such a method will be greatly on the side of the patient. The French call us empirics for this very reason; but we need not be ashamed of the appellation, as long as we can show that, with fewer works on systematic medicine than they can boast of at present, our public and private practice will, nevertheless, exhibit results to which the practitioners of France have, as yet, made but an inconsiderable approach. It will be my study, while I conduct this Journal, to prove, to the full extent of its import, the meaning of this assertion, by some such evidence as our ingenious, and certainly very learned, brethren beyond the channel shall not have reason to complain of.

A long residence among them,—repeated visits to their welladministered hospitals,—the knowledge I have endeavoured to acquire of their character and practice, (both, I confess, very respectable,)—and the various professional occasions I have had of coming in contact with them,—together with a reference to their own published results,—are the grounds on which I am willing to rest the strength of my future demonstrations.

If it be admitted, therefore, that facts and practical observations are the best means of arriving at truth in the investigation of diseases; it follows that we cannot have too many such facts. It is precisely on this account that I venture to submit to the public the following very recent case, taken from my note-book.

On the 22d of August last, I was pressingly summoned to the bed-side of Mrs. N—, whom I found suffering from incessant sickness, attended by a most excruciating pain at the pit of the stomach. She had, for some weeks previously, complained of being bilious, but no medical assistance had been deemed necessary. The present attack came on five days before my first visit, and, although in its effects it appeared not to differ from those she had before experienced, its severity was so much greater, that an apprehension of danger was entertained by a female friend with whom the patient lived. The symptoms becoming at last very urgent, medical aid was procured.

The patient was stated to be about forty years of age; appeared to have a considerable *embonpoint*, and was the mother of three children. Weighed down by domestic afflictions, she had, at last, become exceedingly low and nervous. During the few months anteceding her present attack, I had been in the habit of seeing her occasionally on private business; and, but a few weeks before it, her whole appearance denoted perfect health: she only complained of a very obstinate constipation of the bowels, which with her, however, had been habitual.

On my arrival, I found her considerably agitated, breathing with great difficulty, sighing deeply, and oppressed by flatulence. She had a staring look; her extremities were cold; the pulse was small and fluttering, but not intermittent,—hard, but not full. Every part of the body was covered with a clammy perspiration. The countenance indicated extreme inward suffering, and was haggard and pale. With difficulty she articulated the few expressions intended to convey the nature of her complaint, to the seat of which she endeavoured more forcibly to direct my attention, by pointing with her finger to the pit of the stomach; while, in so doing, she would raise herself on her elbow, soon to sink again exhausted, and almost breathless, on her pillow.

I begged her to be composed, and to lie quiet on her back, while I attentively examined the part. Pressure augmented the pain; and, while employing this method of exploration, I became sensible of something like an enlargement of the liver.

At this juncture, the patient was suddenly seized with vomiting, and brought up a large quantity of a greenish-brown viscid fluid, not without many painful efforts. A considerable quantity of the same matter, ejected from the stomach during the few hours which preceded my visit, was shown to me at the same time; and I was informed that a much larger quantity had been vomited in the course of the three previous days. The tongue appeared dry, and of a reddish-brown colour. The bowels had not acted for two days, notwithstanding a great deal of medicine, which the patient had taken of her own accord, with a view to relieve them.

The thirst she felt was unquenchable; yet, when she drank any liquid, however simple, she felt as if "it scorched her throat and stomach." For some days she had taken no nourishment, with the exception of a little tea, which she no sooner swallowed than it was again rejected.

Questioned as to any pain in any other part of the body, she assured me she felt none, and had *never felt* any before. Her earnest prayer, in the present instance, was for an anodyne draught.

To render the case still more complicated, Mrs. N. declared herself to be in the seventh month of her pregnancy; and was deeply involved, at the time of the attack, in a law-suit of doubtful issue.

I desired twenty ounces of blood to be taken immediately from the arm, a large blister to be applied on the epigastrium, and mucilaginous drinks, with occasional saline draughts, to be given in the course of the day. A composing mixture was ordered for the night; and an enema was directed to be applied immediately, and to be repeated two hours afterwards.

At my next visit, I found the blood which had been extracted to exhibit the inflammatory, though by no means a very thick, crust. The coagulum was very abundant. The serum, on the contrary, was both scanty and dense, and had a curious appearance of an oily opalescence. The blood had stood but a few hours when I examined it. The patient expressed herself relieved: she had suffered less from pain, as well as from sickness; seemed more composed, and received my cautious suggestion of settling her worldly affairs, ere the complaint *assumed a more serious turn*, with an assenting smile. The blister had done its duty but partially. The first lavement had been retained; the second brought away highly-offensive, liquid, and bilious stools. Fifteen leeches were now ordered to the epigastrium, and the following was prescribed to be taken in the course of the day:

℞. *Decoct.* lichenis, f. ℥viii.

Liquoris antimonii tartarizati, f. ℥i.

Liquoris ammoniæ acetatis, f. ℥ii.

M. et cochlearia tria tertiis horis ægrotæ exhibentur. The patient was moreover desired to drink abundantly of a weak lemonade, with nitrate of potash and the mucilage of quinces. The reader will readily perceive the object of these various medicines and applications.

Subsequently to the first bleeding, and particularly after the first evacuations in consequence of the enemas, the pulse acquired a great development, and beat with more freedom and regularity. The lower extremities became rather warm, the hands remaining still chilly and clammy. The patient complained of an insufferable intestine heat. I now ventured to exhibit a few grains of calomel, with James's powder and the cathartic extract.

In the evening I was sent for hastily, when I found that the patient had been delivered, after a few minutes of labour-pain, of a small dead fetus, bearing every mark of having been long dead. The funis was livid, and in a putrescent state; the skin peeled off readily from the body.

During the night, there seemed to be a total cessation of both pain and sickness for a while. The leeches had relieved her much. Still the sensation of a burning heat in the throat continued when she swallowed any liquid; and the action of the calomel excited a slight uneasiness in the bowels previous to its operation.

On the following, or third day, I found my patient completely exhausted. She had had no sleep, notwithstanding the opiate draught. Fever had supervened; and the pain, tension, uneasiness, and flatulence at the stomach, had returned. There was much anxiety depicted in the countenance. The patient started up in bed several times, from mere restlessness, to arrange and move her pillows in various ways. Her mind was collected and calm. She now despaired of her own case. I gave a few directions, repeated my former unfavourable prognostic to her friends, and took my leave. The poor sufferer quitted this world not many hours afterwards. "Ob id etiam, (as Morgagni, on a similar occasion, with much simplicity, observes,) vitâ, multo longiori, digna quod mandavit moriens; id quod perpaucæ mulieres facerent, ut diuturni sui vomitus causa per anatomen quæreretur."

The cavity of the abdomen was the only one I examined. Mr. Wade, the very intelligent apothecary of the Westminster General Dispensary, assisted me on the occasion. I had summoned my friends Dr. Hutchinson and Mr. H. C. Hutchison, but the early hour at which I was forced to operate prevented them from attending.

No outward sign betrayed the existence of any inward disease, save a little swelling immediately below the false ribs. The body was in volume, colour, and firmness, perfectly natural. On the abdominal parietes being cut into, divided, and laid aside, the first object which attracted our attention was the liver occupying a much larger space than in ordinary cases, of an unhealthy colour, approaching that of a mixture of milk and coffee. This viscus extended as far as the left hypochondrium, within the cavity of which the free margin of the left lobe appeared to have penetrated deeply, so as to come in contact with the spleen: it thus concealed from our view both the stomach and the transverse colon. On being removed from its attachments, the stomach offered an extensive blush of inflammation on its external surface: when cut into, large patches of the mucous membrane were found to be in a very inflamed condition, particularly near the cardia, where the muscular coat of the stomach, when separated from its interior and external coverings, partook of the same condition in a high degree. In two distinct parts of the large curvature, and nearer to the left pouch, there were evident signs of approaching gangrene; and, in a third spot, perforation of the mucous and muscular coats had already taken place. The peritoneal covering was still intact. The whole intestinal canal had a very dark greenish tinge, and was abundantly moistened by a thick oily fluid, of a yellowish-green colour, having a bitter and nauseating taste. The intestinal tube was examined minutely, and found to have been inflamed in various places, particularly the cœcum; but this state of the mucous membrane of the intestines was deduced more from the highly-injected condition of its blood-vessels, than from any red colour of its general surface, which the presence of an abundant flow of bile every where precluded us from seeing, in consequence of that fluid having imparted its peculiar colour to the inner membrane.

Every other viscus of the abdomen was examined in succession, including the uterus and its appendages, and found to be healthy. The latter, of course, partook of those usual appearances which denote recent parturition. The mesentery was most beautifully injected. The omentum exhibited no unhealthy appearance.

The liver, which had before been laid aside for farther examination, was now again submitted to our inspection. It presented altogether, when completely detached from the body, a larger mass than I had been in the habit of seeing, during the numerous necroscopic inspections I have had occasion to attend.

We conjectured that it might weigh about twelve pounds. On making a small incision into its peritoneal covering, and raising one of the divided sides of that membrane, the whole was easily detached from the liver, without any effort or laceration. The appearance of this transparent membrane was most beautiful; there were no injected vessels in it. On endeavouring now to make a pressure, even gently, with the finger, on the surface of the liver, its parenchyma gave way, and the finger penetrated quickly within it, forcing out, at the same time, a copious flow of a fluid similar to that which we noticed on the internal surface of the intestines. The same occurred in every other portion of the liver. In fact, this viscus seemed to have been as if macerated in bile, and exhibited, in the most complete manner, the appearances of the *foïe gras* of geese and other birds, the liver of which is made to swell to an enormous size by the skilful hand of the epicurean.

On coming afterwards to that part within which the gall-bladder is lodged, we were struck, as we thought, with a want of the latter. In this supposition we were mistaken. The gall-bladder was indeed *in situ*, but not as we are wont to find it, membranous, translucid, containing its natural fluid, &c. In the present instance, this receptacle of the bile presented a thick carneous body, the half of which contained, within a narrow cavity, a very small quantity of a light-yellowish transparent fluid, wholly soluble in warm water; while the remaining portion was distended, and closely embraced a calculus of a spheroid form, flat, and measuring one inch in its principal diameter. The orifice of the cystic duct was contracted, scarcely admitted the introduction of a small probe, and was effectually closed from within by one of the extremities of the calculus. Of the nature of the latter I shall speak more definitely when I shall have subjected it to a proper analysis. In its external characters, it appeared to be of a pale or dusky yellow colour, light, rough at its elongated extremities, having crystallized depositions on a few points of its surface. The appearance of the latter gave one the idea of the body of the calculus being formed of an assemblage of small globules of an earthy nature.

The accompanying diagrams are intended to convey a portrait of the above appearances; both parts being represented nearly of their natural size.

*A, Morbid appearance of the fleshy gall-bladder, containing the calculus, B, seen through the longitudinal incision, marked by the dotted lines.*

C. *External appearance of the calculus.*

I have shown the preparation to several of my friends, and, among others, to Sir E. Home, Dr. Macleod, and Mr. Cliff, of the College of Surgeons; where it will be deposited among their very interesting collection of calculi, which have lately been most judiciously arranged and displayed.

The various ducts exhibited no deviation from their natural arrangement.

My medical conviction in this case is, that the excess of bile, which the common receptacle for that fluid could no longer receive, from the presence of the calculus, was occasionally thrown, in its undiluted state, into the intestines and stomach; where, having for some years produced all the unpleasant symptoms which Mrs. N. experienced at different times, had at last been the cause of that inflammatory state of the mucous membrane which proved fatal. Another, and a natural, consequence of such a combination of circumstances, has been to soften down the parenchyma of the liver, to increase its volume, and, by producing constant irritation, to excite that organ to an augmented secretion of bile.

One other remark I may make, and that is, that, wherever the bile seems to have been stagnant for a time, a considerable production of fat has been the consequence. I have seldom witnessed the abdominal viscera loaded with so much adipose substance.—A. B. G.

## Figures and Tables

**Figure f1:**
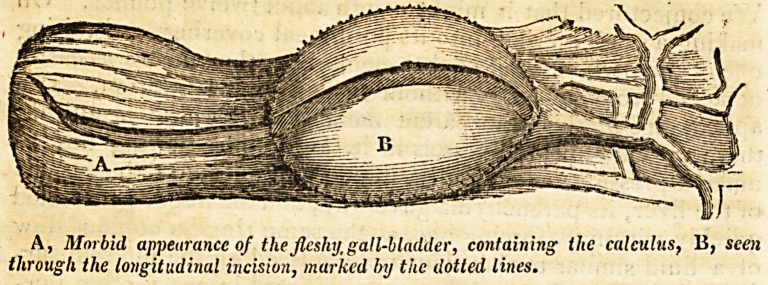


**Figure f2:**