# Errata in Last Number

**Published:** 1821-11

**Authors:** 


### ERRATA IN LAST NUMBER.

Page 368, line 9 from the bottom, for *those* read *the.*

—— 369, line 7 from the top, for *make a pressure* read *make pressure.*

